# Production of small ruminant morbillivirus, rift valley fever virus and lumpy skin disease virus in CelCradle™ -500A bioreactors

**DOI:** 10.1186/s12917-021-02801-4

**Published:** 2021-02-27

**Authors:** Halima Rhazi, Najete Safini, Karima Mikou, Meryeme Alhyane, Khalid Omari Tadlaoui, Xiangliang Lin, Nandini P. Venkatesan, Mehdi Elharrak

**Affiliations:** 1 Laboratory of functional and environmental ecology, Faculty of Sciences and Technology Sidi Mohammed Ben Abdellah University, Imouzzer Road, BP 2202 Fez, Morocco; 2 Laboratory of Research and Development virology, MCI Animal Health, Lot. 157, Zone Industrielle Sud-Ouest (ERAC) B.P: 278, 28810 Mohammedia, Morocco; 3 Esco Aster, 21 Changi South Street 1, Singapore, 48677 Singapore

**Keywords:** CelCradle™ -500A, PPR virus, RVF virus, LSD virus, Vero cells, LT cells

## Abstract

**Background:**

Animal vaccination is an important way to stop the spread of diseases causing immense damage to livestock and economic losses and the potential transmission to humans. Therefore effective method for vaccine production using simple and inexpensive bioprocessing solutions is very essential. Conventional culture systems currently in use, tend to be uneconomic in terms of labor and time involved. Besides, they offer a limited surface area for growth of cells. In this study, the CelCradle™-500A was evaluated as an alternative to replace conventional culture systems in use such as Cell factories for the production of viral vaccines against small ruminant morbillivirus (PPR), rift valley fever virus (RVF) and lumpy skin disease virus (LSD).

**Results:**

Two types of cells Vero and primary Lamb Testis cells were used to produce these viruses. The study was done in 2 phases as a) optimization of cell growth and b) virus cultivation. Vero cells could be grown to significantly higher cell densities of 3.04 × 10^9^ using the CelCradle™-500A with a shorter doubling time as compared to 9.45 × 10^8^ cells in Cell factories. This represents a 19 fold increase in cell numbers as compared to seeding vs only 3.7 fold in Cell factories. LT cells achieved modestly higher cell densities of 6.7 × 10^8^ as compared to 6.3 × 10^8^ in Cell factories. The fold change in densities for these cells was 3 fold in the CelCradle™-500A vs 2.5 fold in Cell factories. The titers in the conventional system and the bioreactor were not significantly different. However, the Cell-specific virus yield for rift valley fever virus and lumpy skin disease virus are higher (25 virions/cell for rift valley fever virus, and 21.9 virions/cell for lumpy skin disease virus versus 19.9 virions/cell for rift valley fever virus and 10 virions/cell for lumpy skin disease virus).

**Conclusions:**

This work represents a novel study for primary lamb testis cell culture in CellCradle™-500A bioreactors. In addition, on account of the high cell densities obtained and the linear scalability the titers could be further optimized using other culture process such us perfusion.

## Background

Animal vaccination is an important way to minimize the spread of diseases that cause enormous damage to livestock, leading to a substantial economical impact. Viruses can be successfully contained by a well-organized vaccination, using sufficient coverage and effective vaccines, which prompted pharmaceutical industries to seek for flexible, cost efficient and operative production technology.

Animal vaccine strains are commonly cultured on adherent cells and less frequently on suspension cells for commercial purposes. Adherent cells such as Vero cells are frequently cultured on 2-D systems such as roller bottles and Cell Factories. but all of them include complicated operation and provide low population densities of cells. Furthermore, they are time consuming and involve heavy operations .

Microcarriers such as Cytodex have also been largely used for adherent cells, offering good mixing and oxygen transfer but often resulting in low cell densities due to accumulation of toxic metabolites and a high shear stress. To alleviate the shear stress problem, other reactors have been developed such as hollow fiber [1], packed-bed bioreactors [2] and Wave bioreactors [3]. In general, they have the advantages of good mixing, aeration and nutrient supply, but require an exterior oxygenation system and sophisticated operation skills .

A novel, single-use bioreactor -the CelCradle™ -500A was evaluated in the current study for culture of adherent cells with the aim of cultivating viruses used in vaccine production for a number of veterinary diseases. This bioreactor for adherent cell culture has been reported to be used successfully for the production of Adeno-Associated Virus [4], Japanese encephalitis virus [5], insect cells for baculovirus production [6], bovine Herpesvirus-1 vaccine [7] and influenza virus [8]. It has also been used for culture of mammalian cells such as HEK 293 for production of G-protein [9], rat pancreatic duct-derived stem cells for insulin production [10] and CHO cells [11]. Previous studies have reported satisfactory results in terms of large surface area, low shear stress and good aeration thus enabling a high cell density and consequently high virus titers. The unique 3D environment provided by the matrices on which cells grow mimics a physiological environment for cell growth.

In this study, we evaluated the use of the CelCradle™ -500A bioreactor for the cultivation of Small ruminant morbillivirus or peste des petits ruminants (PPR), Rift valley fever (RVF) and Lumpy skin disease of cattle (LSD) viruses using two types of cells; Vero cells for PPR cultivation and RVF viruses and primary lamb testis cells (LT) for LSD virus.

PPR is a contagious viral disease of goats and sheep often associated with high morbidity and mortality [12]. Prophylactic administration of a live attenuated vaccine provides strong immunity and is the best method of disease prevention. The RVF virus (family of *Phenuiviridae*) causes a disease transmitted mainly by mosquitoes with potentially severe symptoms among both humans and animals. RVFV is an enveloped RNA virus characterized by a genome composed of three segments designated L, M and S of negative or ambisense polarity [13]. An effective way to establish solid herd immunity is through regular vaccination. Lumpy skin disease of cattle is caused by a DNA virus belonging to the *Poxviridae* family, *Capripoxvirus* genus. LSD is an acute contagious disease causing great economic losses due to skin damage, reduced milk production, mastitis, lowered fertility, and sometimes death due to secondary bacterial infections. Vaccination confers animals with a long-lasting immunity [14, 15].

## Results

### Cells growth kinetics

**Fig. 1:**
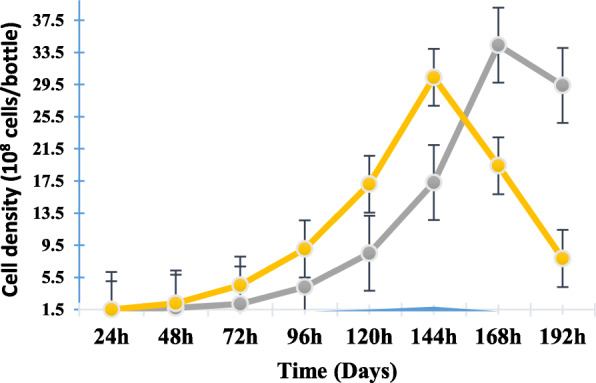
Kinetics of Vero cell growth in medium supplemented by 5% (5F, blue), 3% (3F, grey), and 1% (1F, yellow) FBS in the CelCradle™ - 500A

To minimize FBS percentage in growth medium for the culture of Vero cells, cells wer cultured in DMEM with different percentages of FBS, (1,3 and 5%) for a total of 168 h. The seed obtained from T-flasks (525 cm^2^) was used to inoculate a CelCradle bottle at a cell density of 1.5 × 10^8^ cells/bottle in each of these culture medium. After 24 h of incubation, the highest cell attachment was achieved in 1% FBS medium with a total of 2.3 × 10^8^ cells/bottle, followed by 2.1 × 10^8^ cell/bottle in 5% FBS, and 1.7 × 10^8^ cell/bottle in 3% FBS (Fig. 1) at 3 h post-seeding. The optimal cell density was achieved at different time points of the culture as shown in Fig. 1.

**Fig. 2:**
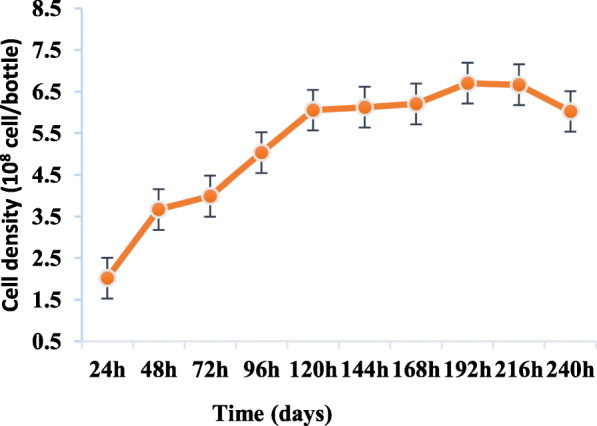
Kinetics of LT cell growth in medium supplemented with 10% FBS in the CelCradle™ - 500A

The kinetics of LT cell growth in the CelCradle™ -500A system over a period of 10 days is represented in Fig. 2. 2.0 × 10^8^ cells/bottle was seeded at day 1 (D1), Peak cell densities were observed at Day 7 of culture with a total nmber of 6.7 × 10^8^ cells/ bottle and a drastic drop in cell numbers there after.

A comparison is made between cell growth from cultures in conventional CF s and the CelCradle™ -500A system (Table 1). In cell factories, the seeding density was 40,000 cells/cm^2^ for both cell types. The cell density at harvest after 4 days/6 days post-seeding was 150,000 cells/cm^2^ and 98,000 cells/cm^2^ for Vero and LT cells respectively. This represents a 3.7-fold and 2.5-fold increase in cell numbers as compared to seeding. Vero cell growth increased by 19-fold in the CelCradle™ -500A as compared to the seeding density whereas LT cells showed a modest increase of only 3 –fold.

**Table 1:** Comparison of cell densities between Cell Factory and CelCradle™ -500A culture systems

	Vero cells		LT cells	
	CelCradle™-500A	Cell Factory	CelCradle™-500A	Cell Factory
Inoculum (cell/ cm^2^)	10,000	40,000	15,000	40,000
Harvest (cell/ cm^2^)	192,000	150,000	43,000	98,000
Incubation time (days)	5	4	8	6
Fold-increase in cell numbers	× 19	× 3.7	× 3	× 2.5
Cell doubling time (h)	28 h	24 h	86 h	76 h
Medium volume (ml/cm^2^)	32 ml	237 ml	32 ml	237 ml

Vero cells had a doubling time (DT) of 24 h in Cell Factories versus 28 h in Celcradle system. For LT cells the DT was 76 h in Cell Factories and 86 h in Celcradle system.

### Virus growth kinetics

#### PPR virus

**Fig. 3:**
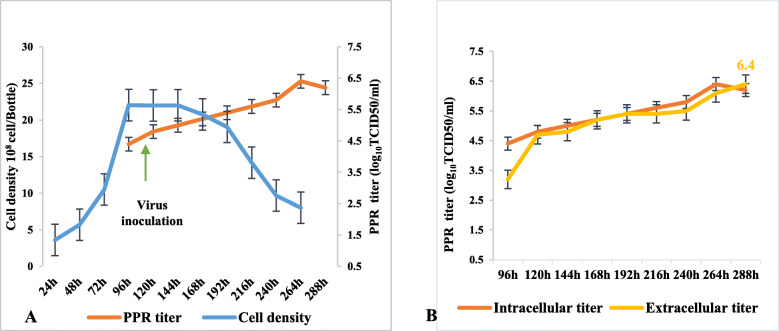
**a** Kinetics of Vero cell growth in 1% FBS-containing media and PPR virus titer. **b** Comparison of titers between extracellular and intracellular virus

As described previously, Vero cells grew optimally in medium containing 1%FBS. This condition was used for all subsequent experiments. Vero cells were inoculated when total cell numbers were 22 × 10^8^ cells/bottle at D4 with PPR virus as described previously (Fig. 3a).

For the next 3 days, cell numbers did not increase (21.99, 22.01, and 22.03 cells/bottle). PPR virus titers however started to increase (from 4.4 to 5 log_10_TCID50/ml) as represented in Fig. 3a below. At the time when cells start excreting virions, there was a drastic drop in cell numbers decreases 8 × 10^8^ cells/bottle. In Fig. 3b we compared titers of the total extracellular and intracellular virus. There is a small difference between the two titers, 4 to 5 days post infection.

#### RVF virus

**Fig. 4:**
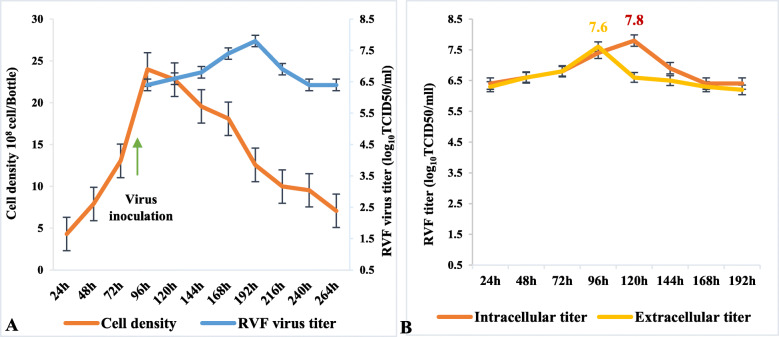
**a** Kinetics of Vero cell growth in 1% FBS –containing media, and RVF virus **b** Comparison of titers between extracellular and intracellular virus

Figure 4a represents kinetics of RVF virus growth on Vero cells during 11 days of cultivation. Cells were inoculated with the virus when total cell numbers were 24 × 10^8^ cells per bottle. As expected, cell numbers drop after inoculation with the virus as soon as 24 h post-innocultion and at day 5 show a drastic drop in cell numbers with a corresponding increase in viral titer. Figure 4b represents the titers of the total extracellular and intracellular virus. After 4 days, the extracellular virus had a titer of 7.6 log_10_TCID50/ml and intracellular virus of 7.8 log_10_TCID50/ml after 5 days of inoculation.

#### LSD virus

**Fig. 5:**
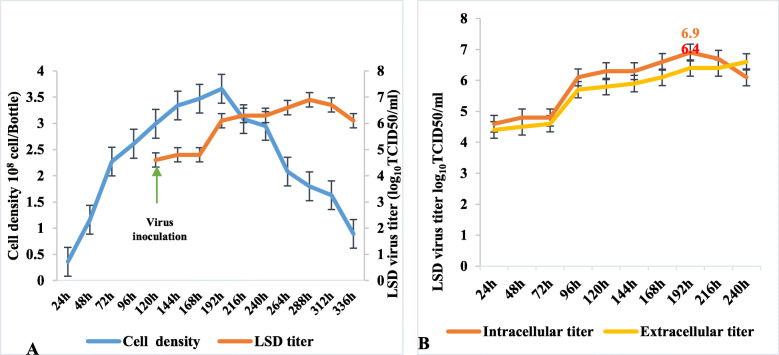
Kinetics of LT cell growth in 10% FBS containing media and LSD virus titers B. Comparison of titers between extracellular and intracellular virus

Cells were inoculated at day 5 with the LSD virus at an MOI of 0.01 and when total cell numbers were 2.2 × 10^8^ cells/bottle. At 4 dpi, LT cells reached their maximum cell concentration of 3.6 × 10^8^ cells/bottle (Fig. 5a). The cells started to secrete LSD virions into the extracellular environment at this point. At D8 post infection, cells secrete the most number of virions as reflected by viral titers (6.9 log_10_TCID50/ml) while cell densities dropped to 1.8 x10^8^cells/bottle.

At day 8, extracellular virus titer was 6.4 log_10_TCID50/ml whereas intracellular virus is 6.9 (6.6 log_10_TCID50/ml) (Fig. 5b).

Table 2 is a comparison of the titers of PPR, RVF and LSD viruses obtained by cultivation in 2 culture systems. In Cell Factories, the PPR titer was 6.3 log_10_TCID50/ml after 5 days of incubation, LSD was 6.5 log_10_TCID50/ml after 5 days of incubation and RVF was 7.8 log_10_TCID50/ml after 4 days of incubation. In CelCradle™ - 500A bioreactor,the PPR titer obtained was 6.4 log_10_TCID50/ml after 7 days of incubation, and LSD virus titers were 6.9 log_10_TCID50/ml after 8 days and 7.8 log_10_TCID50/ml for RVF after 5 days of incubation.

**Table 2:** Comparison of viruses titers and incubation time using Cell Factories and Celcradle systems

Viruses	Incubation period (Days)		Virus titer (log_10_TCID50/ml)		Cell-specific virus yield (virions/cell)	
	CelCradle™-500A	Cell Factory	CelCradle™-500A	Cell Factory	CelCradle™-500A	Cell Factory
PPR	7	5	6.4	6.3	1.29	6.33
RVF	5	4	7.8	7.8	25	19.9
LSD	8	5	6.9	6.5	21.9	10

## Discussion

Peste des Petits Ruminants, Rift Valley fever and Lumpy skin disease are among the most frequent and devastating diseases of livestock in Africa and Asia [12, 13, 15]. Those diseases can only be controlled by preventive measures through vaccination. As for RVF, vaccination is necessary not only to immunize animals but also to prevent animal to human transmission.

Most of the veterinary vaccines are produced in adherent cells; Vero cells are the preferred substrate for PPR and RVF viruses, and LT primary cells are the preferred substrate for LSD viruses [16–18]. Currently, these cells are cultured in open systems using Cell factories or roller bottles, which lead to a high risk of contamination. These systems have the added disadvantage of a large footprint and are labour -intensive as well. The conventional CF and roller bottles also involve lengthy handling operations, in addition CF provide poor oxygen transfer and present surface area limitations leading to low cells densities [9, 19]. Therefore, the development of an efficient adherent cell culture process is desirable.

Various types of bioreactors have been used for adherent cells like hollow fiber, packed-bed and disposable Wave bioreactors. They offer good oxygenation but they necessitate sophisticated skills to operate. In this study we evaluated the CelCradle™ -500A system, for the production of LSD vaccine on LT primary cells and RVF and PPR vaccines on Vero cells. Our evaluation was based on (i) the comparison of the cell growth of the two type of cells in the CelCradle™ -500A and Multitrays (ii) comparison of viral titers of the 3 respective viruses obtained in both systems.

To reduce serum percentage in growth medium for the culture of Vero cells, cells were cultured in DMEM with different percentages of FBS, (1,3 and 5%). the most important cell attachment was achieved in 1% FBS medium with a total of 2.3 × 10^8^ cells/bottle after 3 h of incubation.

Cell gowth kinetics were evaluated for the 2 different cell types using conventional Cell Factories and the CelCradle™-500A. For Vero cells, a 19-fold increase in cell growth as compared to seeding density with a corresponding decrease in doubling time was observed. This is advantageous from the point of scale-up strategies and to obtain higher virus titers as a results of high cell densities.

The CelCradle thus facilitates an enhancement in cell proliferation as compared to conventional culture system such as the CF. In a similar study carried out in Japan with Vero cells by Hiroko et al. (2007), the author reported a total cell number of 28 × 10^8^ cells/bottle of 7 days of incubation as compared to 30 × 10^8^ cells/bottle in 5 days in our study [5]. Different types of microcarriers were used for Vero cells cultivation achieving a lower cell concentration; 1.35 × 10^5^ cells/ml, 1.55 × 10^5^ cells/ml, 2 × 10^5^ cells/ml, 2.40 × 10^5^ cells/ml, 4.7 × 10^5^ cells / ml, 5 × 10^5^ cells/ml, 1 × 10^6^ cells/ml, 1.85 × 10^6^ cells/ml, and 2.6 × 10^6^ cells/ml [20–23].

Typical cell doubling time of Vero cells is 24 h [24], which given by CF in our study. On CelCradle™ -500A system we obtained a DT of 28 h. which is consistent with a relatively longer cell doubling time of 28–38 h in a study carried out by Yang et al. to examine Vero cells bead to bead transfer in spinner flasks with microcarriers. Therefore, it appears that, when the Vero cells were cultured on Cytodex, the doubling time of Vero cells was usually longer than 24 h [25]. In addition, Lai et al. (2019) reported a doubling time for Vero cells of 44.4 h using the Celcradle system which proves the efficiency of our cell growth conditions [8].

LT cells were grown in the CelCradle™-500A to a total number of 6.7 × 10^8^ cells/bottle after 8 days of culture; with CF’s, we obtained 6.23 × 10^8^ cells/bottle after 6 days of culture. The CelCradle therefore yields a few higher cell numbers than the CF’s. To date, very few studies have reported efficient cultivation of primary cells using the CelCradle™ -500A system. Chen et al. in (2016) differentiate rat pancreatic duct- derived stem cells (PDSCs) and successfully obtained after 10 -fold increase in cell density at the end of the culture period of 7 days [10]. In 2007 S. Frauenschuh et al. cultivated primary mesenchymal stem cells on Cytodex microcarriers given a cell concentration of 10,000/14 ml [26].

The CF has an added disadvantage in that after 6 days of culture, cell numbers dropped drastically and showed a steep decrease in viability. This is due to pH regulation difficulty, low aeration and metabolites limitation. Such problems were not encountered in CelCradle™-500A cultures.

In this work, we studied kinetics of three viruses on two different production systems. The obtained viral titers were similar in both systems for PPR and RVF viruses. As for LSD virus, the titer was higher in CelCradle™ - 500A as compared to CF by 4.78 × 10^6^ infectious units of virus/ml. The run time was one to 3 days longer in CelCradle™ -500A than in CF, it was also reported to be 6 days longer by Lewis Ho et al. in 2004, where it was shown that the cultivation of HEK 293 cell line using Celcradle system took 12 days using Celcradle system versus 6.5 days using Cell Factories system [9].

The virus characteristics and impact on the cell substrate was variable in the 3 viruses cultivated in the CelCradle. PPR and RVF inoculation block cell growth immediately after infection whereas after LSD virus infection, cells continue their growth for 2 days before a drop in viability. This can be explained by the fact that LSDV is an intracellular virus with slow replication as compared with PPR and RVF. For the 3 viruses, we observed that intracellular titer is higher than the extracellular one. This could be explained by the fact that the virus in the supernatant is diluted in the medium.

Conventional culture systems for adherent cells tend to reduce cell growth because of their space limitation and design. The CelCradle™-500A system offers many advantages at different levels. The 3D environment increases surface area for culture, a uniform distribution of cells and maximum aeration and nutrition by virtue of the “Tide motion” principle. This provides low shear stress, high aeration with no O_2_ limitation, and a foam-free culture environment. Thus, optimum cell density and consequently, high viral titers are possible. Moreover, macrocarriers –the matrices for cell growth allows the adherence of Vero cells in low serum medium, which reduces the cost of the production.

Owing to the simple design, this system is extremely easy to handle and operate and can be used for small- scale vaccine production and for preparation of seed trains. These benefits enable it to become a simple and economical system for high-density cell culture and virus production. It has been successfully utilized to grow primary cells, and to produce PPR, RVF and LSD vaccines.

Viral titers were comparable in both systems. Since all our trials were done without medium replenishment, these titers could be optimized by the usage of a perfusion system, and this being a continuous system could enhance volume of virus harvested and possibly the titer as well.

## Conclusion

Taking into consideration that the CelCradle™ -500A is a laboratory scale bioreactor which yields titers equivalent to Cell factories that are conventional industrial scale systems, this represents a promising preliminary study for potential use of a novel bioreactor. It important to mentionne that cultures in the the Tide motion bioreactors are linearly scaeable and production of the 3 viruses reported here can be readily scaled up in TideXcell 2-5000 L bioreactors.

## Methods

### Cells and viruses

Vero cells were purchased from ATCC (no. CCL-81) and initially cultivated in 1, 3 or 5% of foetal bovine serum (FBS)-containing DMEM medium for the purpose of selecting the optimal conditions for further cell cultivation and virus production experiments. Primary lamb testis cells LT, were obtained by castration of a healthy 3 month old male and obtaining cells from the testis. Cells were prepared and propagated in Dulbecco’s modified Eagle’s medium supplemented with 10% FBS. Three attenuated vaccine strains were used in this study: PPRV Nigeria 75 strain [27], RVF Clone 13 T virus [28] and LSD virus Neethling strain [29].

### Bioreactor system

The CelCradle™ - 500A is a single-use bioreactor capable of yielding high-density cell cultures for production of vaccines, recombinant proteins and monoclonal antibodies. The bioreactor used in this study consists of two compartments; an upper chamber made of polyethylene terephthalate containing 5.5 g of macrocarriers which provide the matrices for cell adherence and growth and a lower compressible chamber (LCC) of low-density polyethylene containing the medium. The cap is equipped with a 0.22 μm PTFE filter.

The CelCradle was mounted on a stage and the parameters for cultivation were set using a control unit. The upward and downward movement of the media provides a “Tide motion”. Cells on the macrocarriers thus receive an alternating cycle of aeration and nutrition.

### Cell culture

Macrocarriers were equilibrated in 400 ml of media. Following this, 100 ml of the respective cells was added in to individual CelCradle™-500A bioreactors.

Cell seeding and attachment period was for 3 h. After this period of incubation, 2 macrocarriers were sampled using sterile forceps, fixed with 2 ml of 95% ethanol, stained with 2 ml of Trypan blue, and were observed microscopically to visualise cell attachment. After determining that the cell attachment was more than 90%, the Tide motion parametres were changed to cell cultuvation. The Tide motion parametres of cell seeding and cell cultivation were as follows:

**Table:** 

Tide motion	Rising rate	Top holding time	Down rate	Bottom holding time
**Cell attachment**	2 mm/sec	20 s	2 mm/sec	0 s
**Cell cultivation**	1.5 mm/sec	20 s	1.5 mm/sec	0 s

Cell density on carriers was also evaluated by a crystal violet dye (CVD) nucleus staining method with 2 macrocarriers taken from the bottle. The carriers were incubated at 37 °C with 1 ml of CVD and vortexed every 15 min. A hemocytometer was then used to count the nuclei as a readout for the number of cells.

The cell doubling time was calculated with the following formula:

Cell doubling time (DT) = ln2/μ, Where μ = ln X_n_ - ln X_n-1_ /t_n_ –t_n-1_.

t: time of sampling (hours), X: cell number at t (cells/bottle).

Experiments were executed in 2 phases: Phase 1 and Phase 2 for optimizing cell-growth and virus culture respectively. Initially, the cell growth kinetics was investigated for Vero cells. Optimum FBS concentrations for Vero cell culture was determined using 1, 3 and 5% serum in the medium. The optimal medium was then used for subsequent virus propagation experiments. For phase 1, seeding cell concentration for Vero cells was 11.36 × 10^3^ cells/cm^2^ for each of the 3 types of medium. In the phase 2 of virus production, cell seeding was 22.72 × 10^3^ cells/cm^2^ .

For LT cells, the feasibility of growing them on the carriers was determined before subsequent experiments. Cell concentration used was 1.7 × 10^3^ cells/cm^2.^ This was essential to determine if indeed primary cells could be cultivated on the carriers. For the virus production experiments, cell seeding of LT cells was 2.77 × 10^3^ cm^2.^

**Table:** 

Cell seeding for cell growth experiments (cells/bottle)		Cell seeding for virus production experiments (cells/bottle)	
***LT cells***	***Vero cells***	***LT cells***	***Vero cells***
2.25. 10^7^	15 × 10^7^	3.66 × 10^7^	30 × 10^7^

In parallel as a head-to-head comparison, Cell Factories (CF, Nunc 10 chamber) with a total surface area of 6320 cm^2^, were seeded with 3.99 × 10^4^ cells/cm^2^ LT cells or Vero cells (with 1500 ml of DMEM supplemented with 5% FBS) and incubated at 37 °C with 5% CO_2_.

### Virus production

Culture medium in each of the 3 bottles CelCradles was replaced by 300 ml of DMEM containing 1%FBS. Cells were inoculated with viruses at an MOI of 0.01 on day 4 for PPR and RVF and on day 5 for LSD virus, already adapted to the respective cells. The titers of the seeded viruses were 6,2 (log_10_TCID50/ml) for PPR, 7,5 (log_10_TCID50/ml) for RVF and 6,3 (log_10_TCID50/ml) for LSD.

After a virus adsorption period of 3 h, 200 ml more of medium was added to each of the CelCradles.

The Tide motion parameters were changed to the following:

**Table:** 

Rising rate	Top holding time	Down rate	Bottom holding time
1.0 mm/sec	20 min	1.0 mm/sec	0 s

### Virus titration

Two samplings of the macrocarriers were carried out daily until D10 post infection in order to determinate the virus growth kinetics. Two milliliter of the supernatant culture medium containing secreted virus, extracellular virus, was obtained at each sampling and stored at 4 °C. Two macrocarriers were also sampled for intracellular virus and stored at − 20 °C until titration. The intracellular virus was obtained by lysing the cells by a freeze/thaw cycle.

For virus titration assays, Vero/LT cells were seeded at a density of 110, 000 cells/well and (100 μl) of serially diluted virus was added to each well. After an incubation period of 96 h at 37 °C, the virus titer was determined by IPMA assay as described by Andy et al*,* 2020 [30].

## Data Availability

The datasets used and/or analysed during the current study are available from the corresponding author on reasonable request.

## Abbreviations

PPR: Small ruminant morbillivirus
RVF: Rift valley fever virus
LSD: Lumpy skin disease
LT: Lamb testis
DT: Doubling time
LCC: Lower compressible chamber
CVD: Crystal violet dye
CF: Cell Factory
